# Ancient Dental Instruments

**Published:** 1853-01

**Authors:** 


*Ancient Dental Instruments.*—We received a few days since for the museum of the Baltimore College of Dental Surgery, from Dr. J. L. Levison, of Brighton, England, three old instruments for the extraction of teeth, said by the gentlemen who presented them to Dr. L., to have been taken from the ruins of Pompeii. They have, certainly, an exceedingly antiquated appearance, and consist of a punch with two prongs, a pair of forceps, and an instrument intended for the extraction of teeth perpendicularly.

